# A Simple Mannose-Coated Poly (*p*-Phenylene Ethynylene) for Qualitative Bacterial Capturing

**DOI:** 10.3390/molecules23082056

**Published:** 2018-08-16

**Authors:** Madalitso Tsakama, Xiaochi Ma, Yonghuan He, Weihua Chen, Xiaofeng Dai

**Affiliations:** 1 Institute of Food Science and Technology, Chinese Academy of Agricultural Sciences, Beijing 100193, China; mtsakama@poly.ac.mw (M.T.); tiaotiao@outlook.com (X.M.); heyonghuan@iccas.ac.cn (Y.H.); 2 Key Laboratory of Agro-products Quality and Safety Control in Storage and Transport Process, Ministry of Agriculture, Beijing 100193, China

**Keywords:** poly (*p*-phenyleneethynylene), sugar coated, foodborne pathogen, *E. coli*, fluorescence

## Abstract

A mannose-functionalized poly (*p*-phenylene ethynylene) was rationally designed to achieve selective detection of bacteria. The polymer was constructed as a signaling unit and was modified by attaching aminoethyl mannose using the carboxylic acid group at the end of the linker. Incubation of *Escherichia coli* with the polymer yielded fluorescent bacteria aggregates through polyvalent interactions. The utility of the mannose functionalized polymer to detect *E. coli* expressing functional FimH mannose-specific lectin on their surface was also demonstrated. The sugar units displayed on the surface of the polymer retained their functional ability to interact with mannose-binding lectin. To determine the optimum binding time, we measured the fluorescence intensity of the polymer-bacteria suspension at intervals. Our results showed that binding in this system will reach an optimum level within 30 min of incubation. The polymer’s affinity for bacteria has been demonstrated and bacteria with a concentration of 10^3^ CFU mL^−1^ can be detected by this system.

## 1. Introduction

Contamination of food by pathogenic bacteria remains one of the major threats to human health despite significant efforts that have been undertaken to limit the extent of bacterial foodborne infections [1,2]. The threat posed by foodborne pathogens such as *Escherichia coli* (*E. coli*) can be monitored by a range of different detection methods. Some of these methods enable only the qualitative confirmation for the presence or absence of a pathogen, while others allow also for the quantification of bacterial load. For detection of bacteria, conventional plating and cultivation still remain the method of choice and by far the gold standard for contamination assessments [3,4,5].

Generally, standard microbiological methods are based on traditional culture assays such as pre-enrichment, differential and selective culture media, and serological techniques for the confirmation of bacteria. In such cases, the assay time can take 4–6 days to obtain a result. Although tremendous progress has been made in the formulation of improved media for selective enrichment and identification, there are still some clear drawbacks associated with these techniques. Traditional cultural assays are time consuming, tedious and often labor intensive [4,6,7,8], taking several days, up to weeks, to verify preliminary positive results.

Other important challenges are the capacity to differentiate between live and dead bacteria and to monitor bacterial growth and the ability to detect and monitor microorganisms within the shortest time period [9,10]. As such, fast detection of bacterial pathogens such as *E. coli* remains an important area and will also, in the future, retain its importance in the food industry and microbiological quality control. 

A large number of human pathogens use cell surface carbohydrates as receptors to facilitate cell adhesion [11]. Type 1 fimbriae are adhesion organelles expressed by Gram-negative bacteria, such as *E. coli* [12]. The subunits at distal tips of type 1 fimbriae are able to mediate mannose-sensitive adhesive interactions, while those at other positions are inaccessible to the ligand [13]. Therefore, a number of culture-independent methods for the detection of bacteria have been developed based on this phenomenon. Such methods include pathogen recognition by fluorescently-labeled antibodies [14,15], DNA probes and microarrays [16,17], carbohydrate functionalized nanodots [18], polymerase chain reaction (PCR) [19] and surface plasmon resonance (SPR) [20,21], enzyme-linked immunosorbent assay (ELISA) [22], and fluorescent in situ hybridization (FISH) [2]. 

As much as some of these methods are based on fluorescent intensity changes of the samples, most of them are very expensive to manage and mostly require specialized training and backup [23]. For any newly-developed reagents capable of detecting bacteria, it is imperative that they should have two functions: strong affinity for bacteria and a bright photoluminescence [24]. This is the greater reason conjugated fluorescent polymers have lately emerged as very interesting functional materials, because they are able to act as both recognition and transmission elements in biosensing systems [25]. The facility for acting as molecular antennae, in which one exciton can patrol up to a hundred repeat units, makes conjugated polymers more powerful than small dyes in biosensing studies. In addition, conjugated polymers are capable of supporting multivalent interactions and can be used in minute quantities [26]. 

Fluorescence is widely used in biotechnology and analytical applications because of its extraordinary sensitivity, high specificity, and simplicity. Compared to conventional sensing materials, conjugated polymers possess the advantages of structural and chemical diversity. Worth noting is their flexibility to have their backbones systematically modified by attaching suitable recognition components at well-defined molecular levels [27,28].

Among the conjugated polymers used for fabricating chemical or biological sensors, poly (*p*-phenylene ethynylene) (PPE) coated with carbohydrate units are of particular importance due to their distinctive functional properties. Apart from simulating multiple interactions, they also exhibit a number of ligands on a single polymer chain [29,30]. Compared to other conjugated polymers, PPEs have received a fair amount of attention in chemical or bioanalysis because they exhibit rapid energy migration along the conjugated backbone. Additionally, they are applied to study quenching-structure relationships owing to their good optical response to environmental changes through the relatively free internal rotation of the alkyne-aryl single bond and facile interchain π-aggregation [31]. Fluorescence has been shown to be an extremely valuable and versatile tool for the investigation of biological systems [32,33]. One of the important advantages of fluorescence is that it allows for real-time observation of the labeled molecules in living systems [34]. In addition, fluorescence is simple and requires very small samples. However, meaningful application of PPEs requires that they must be highly fluorescent with cost-effective designs and production protocols.

In view of this, we synthesized a novel mannose functionalized poly (*p*-phenylene ethynylene) (MCPPE) and evaluated its optical properties. The polymer’s ability to be quenched by concanavalin A and the potential to be used in biosensing were equally demonstrated. The polymer which was constructed as a signaling unit was modified by attaching aminoethyl mannose using the carboxylic acid group at the end of the linker. Emphasis was placed on devising a bacterial detection system that is cost-effective and easy to manage for rapid qualitative bacterial detection. This work was thoroughly presented in our earlier communication [35]. In this study, the performance of the (MCPPE) to selectively detect bacteria was comprehensively assessed and this paper gives a detailed description of the of the polymer’s ability to qualitatively detect *E. coli*. In addition, the optimum binding time and the concentration of bacteria that can be detected by this system were also evaluated.

## 2. Results and Discussion

**Scheme 1:**
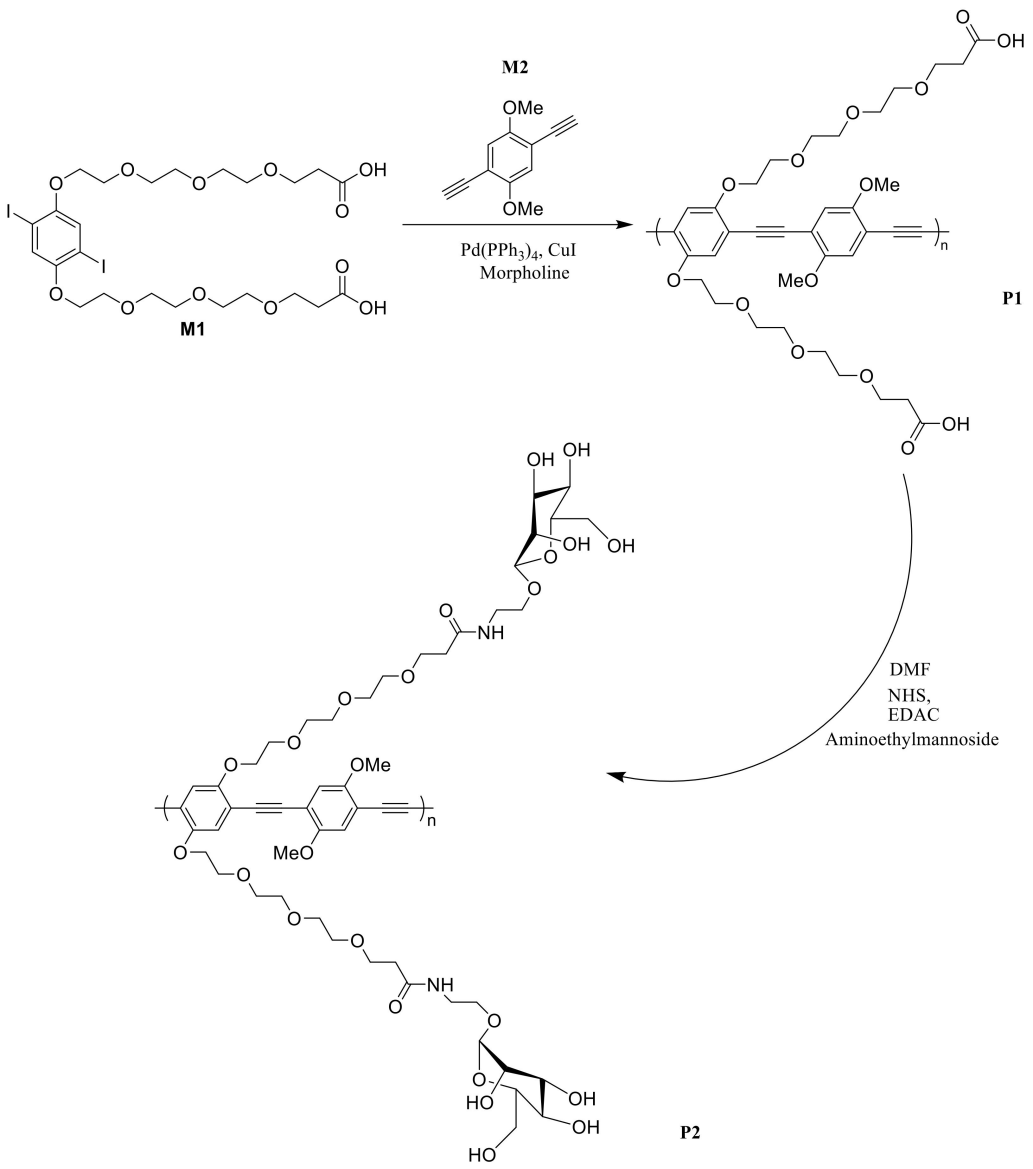
Synthesis of mannose-coated poly (*p*-phenylene ethynylene) P2.

Synthetic work for the sugar-coated polymer (Scheme 1) was first described in our earlier publication [35]. Representative photo-physical data of the polymer are summarized in Table 1. In general, the polymer solution exhibited a blue-green emission and displayed an absorption peak at 393 nm with an emission λ_max_ of 466.5 nm. These spectral properties are identical to those of conversional PPEs in organic solvents. The extent of sugar loading for this polymer was found to be at 30%. Despite the indistinguishable optical properties to other PPEs reported by other groups [29,30,36,37], the polymer exhibited a large Stokes shift which made it a potential candidate for sensing studies.

Small Stokes shifts are associated with serious self-quenching and fluorescence detection error because of excitation back scattering effects. As a result, a large Stokes shift in this case guaranteed reduced auto fluorescence, consequently leading to enhanced sensitivity when applied in biosensing. 

**Figure 1:**
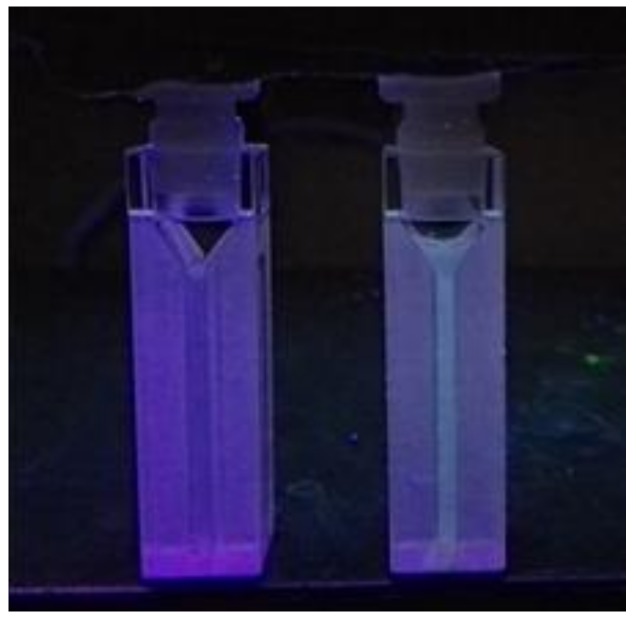
Image of BL21 after incubation with mannose-coated polymer (**right**) against untreated BL21 cells (**left**). Imaging was carried out under a transilluminator.

**Figure 2:**
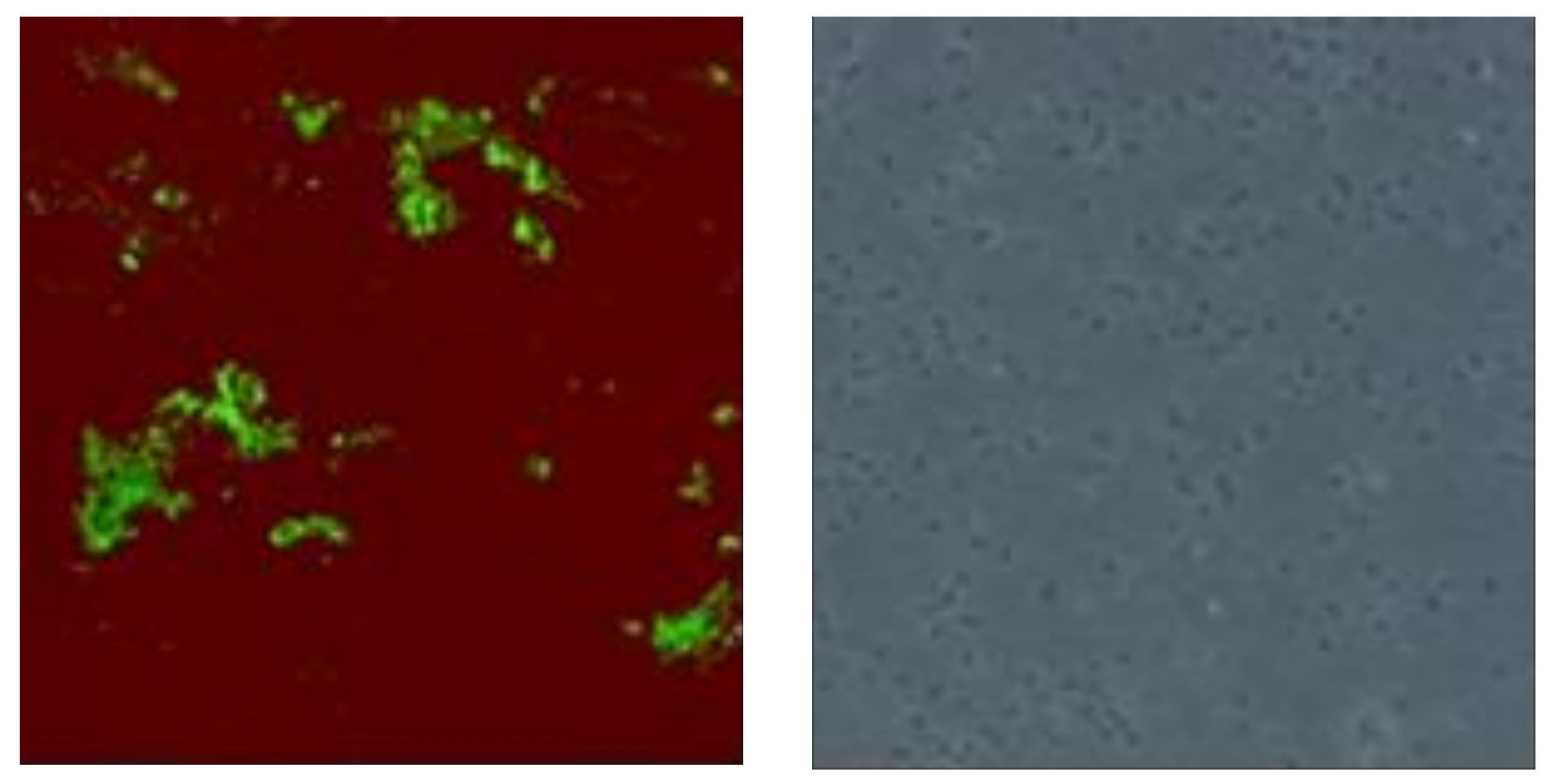
Images of BL21 cells (OD600 = 1) incubated with mannose-coated polymer (**left**) and PBS solution (**right**).

To ascertain the capability of the sugar coated polymer to capture bacteria, we used *E. coli* (BL21), a normal inhabitant of the intestines. We incubated the polymer with an *E. coli* cell suspension for 45 min after which the cells were washed several times using PBS solution and resuspended in PBS. A sample drawn from the cell-PBS suspension showed clustered precipitates that fluoresced under a transilluminator (365 nm) (Figure 1) and when observed under a laser confocal microscope where green precipitates were observed when excited at 405 nm (Figure 2).

This observation signified successful capture of the bacteria by the polymer because *E. coli* cells on their own do not emit fluorescent light at this wavelength. The untreated sample showed no cluster formation or any fluorescence emission from the bacterial cells. As such, we attributed the clusters to the many bacterial cells that complexed with the polymer through polyvalent bonding. Much as type 1 fimbriae mediate mannose sensitive adhesive interactions, hydrophobic interactions between the polymer and the cell wall amino acids are also another way to explain the staining, but this usually to a small extent in cases of this nature.

To establish the optimum binding time for the polymer and bacterial cells, we incubated bacterial cells with the polymer and drew a sample from the polymer-*E. coli* mixture at 5 min intervals. The drawn samples were subjected to fluorescence intensity measurements to determine the extent of fluorescence quenching as a function of time. By plotting time against fluorescence intensity, it was demonstrated that fluorescence intensity of the cell-polymer mixture reduced with time up the 30 min mark where there was no further significant change.

**Figure 3:**
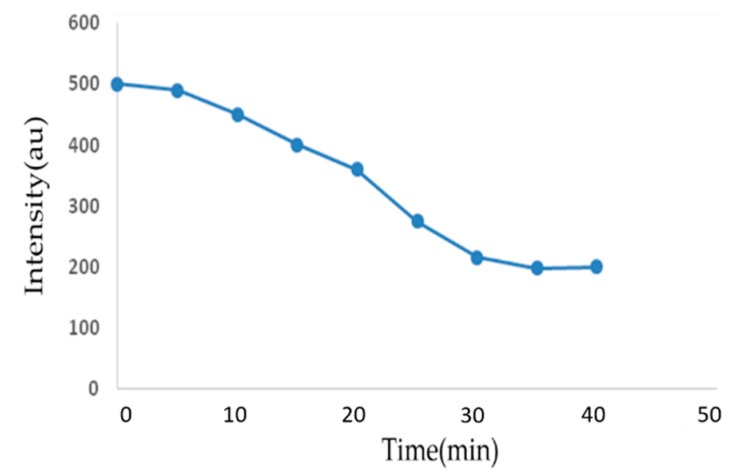
Fluorescence intensity and time plot following progressive binding of bacterial cells to the mannose-coated polymer.

These measurements indicated that the optimum binding occurs within the first 30 min of incubation (Figure 3). As such, for this polymer, incubation period for polymer-bacterial studies has to be a minimum of 30 min.

**Figure 4:**
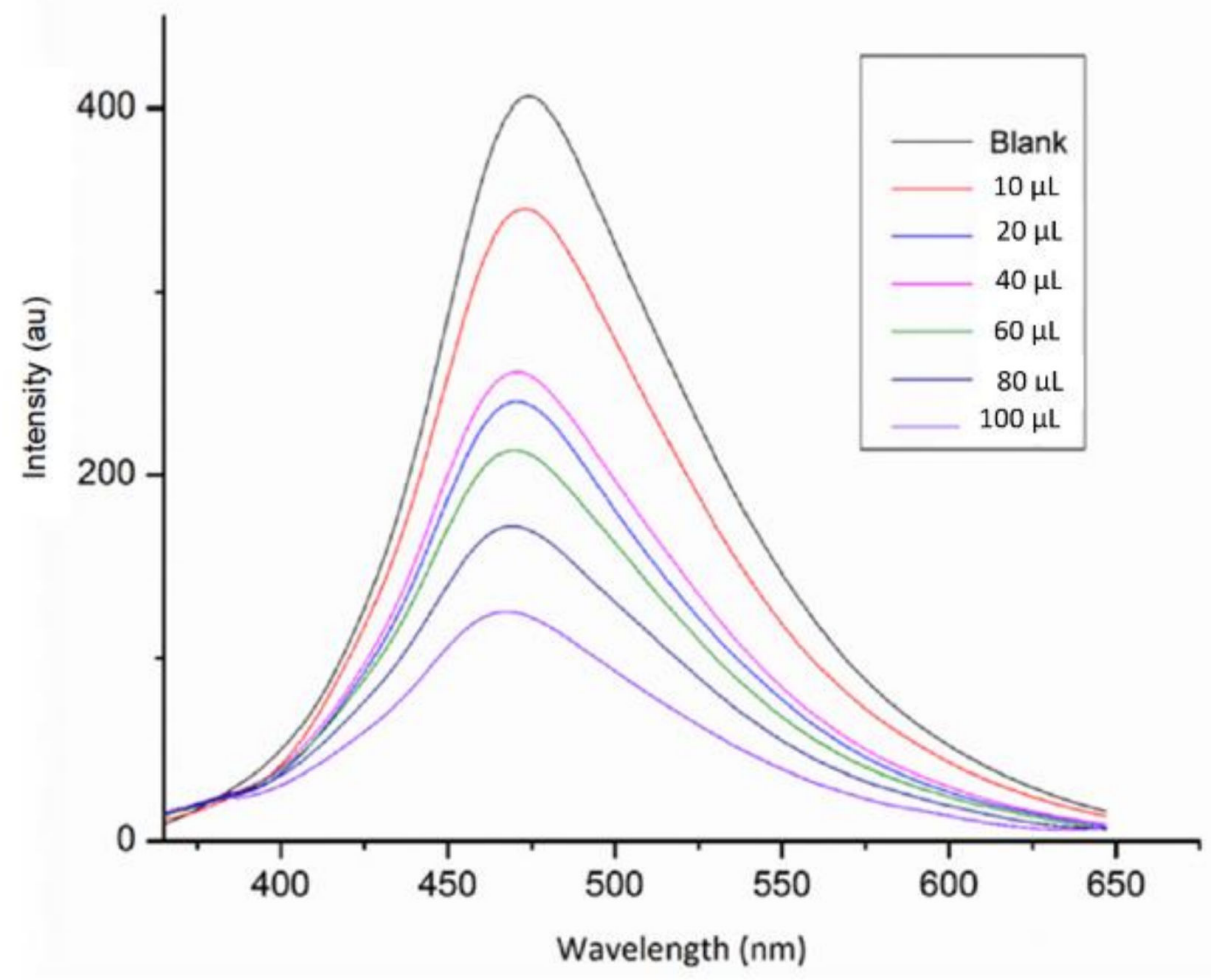
Fluorescence spectra from addition of various concentrations of *E. coli* (OD600 = 1) to mannose-coated PPE.

In our previous study, successful conjugation of the sugar to the polymer and its quenching efficiency were ascertained by determining its fluorescence response behavior to the addition of concanavalin A (Con A) (data not shown) [35]. In the same vein, we set up a similar experiment to assess the effect of incubating bacteria and the polymer at various concentrations and measured the resulting fluorescence intensity. Figure 4 shows the fluorescence spectra from the incubation of *E. coli* and the mannose-coated polymer. Several serialized additions of *E. coli* led to a reduced fluorescence signal. However, no significant shift in the emission maximum was observed, suggesting non-aggregation. The binding of *E. coli* to the polymer was shown to be highly specific and since there was no, or minimal, quencher-induced aggregation, the possible mechanism for the quenched fluorescence intensity could be ground state complex formation due to static quenching [38].

The availability of large numbers of hydroxyl groups on the sugar enables formation of complex networks of hydrogen bonds in which the hydroxyl group acts both as a donor and an acceptor. Hence, cooperative hydrogen bonds are formed between corresponding carbohydrate amphiphilic surfaces and the protein surfaces [39]. In this case, bacterial cells and the mannose-coated polymer formed a ground state complex and, upon excitation, the preformed complex was effectively quenched. Although collisional quenching processes are present, their influence in the fluorescence quenching of PPEs is negligible [37,38]. The behavior of the bacteria-polymer mixture, with regard to fluorescence intensity reduction, provided evidence that the epitopes on the bacterial pili are similar to the mannose-binding receptors of Con A.

**Figure 5:**
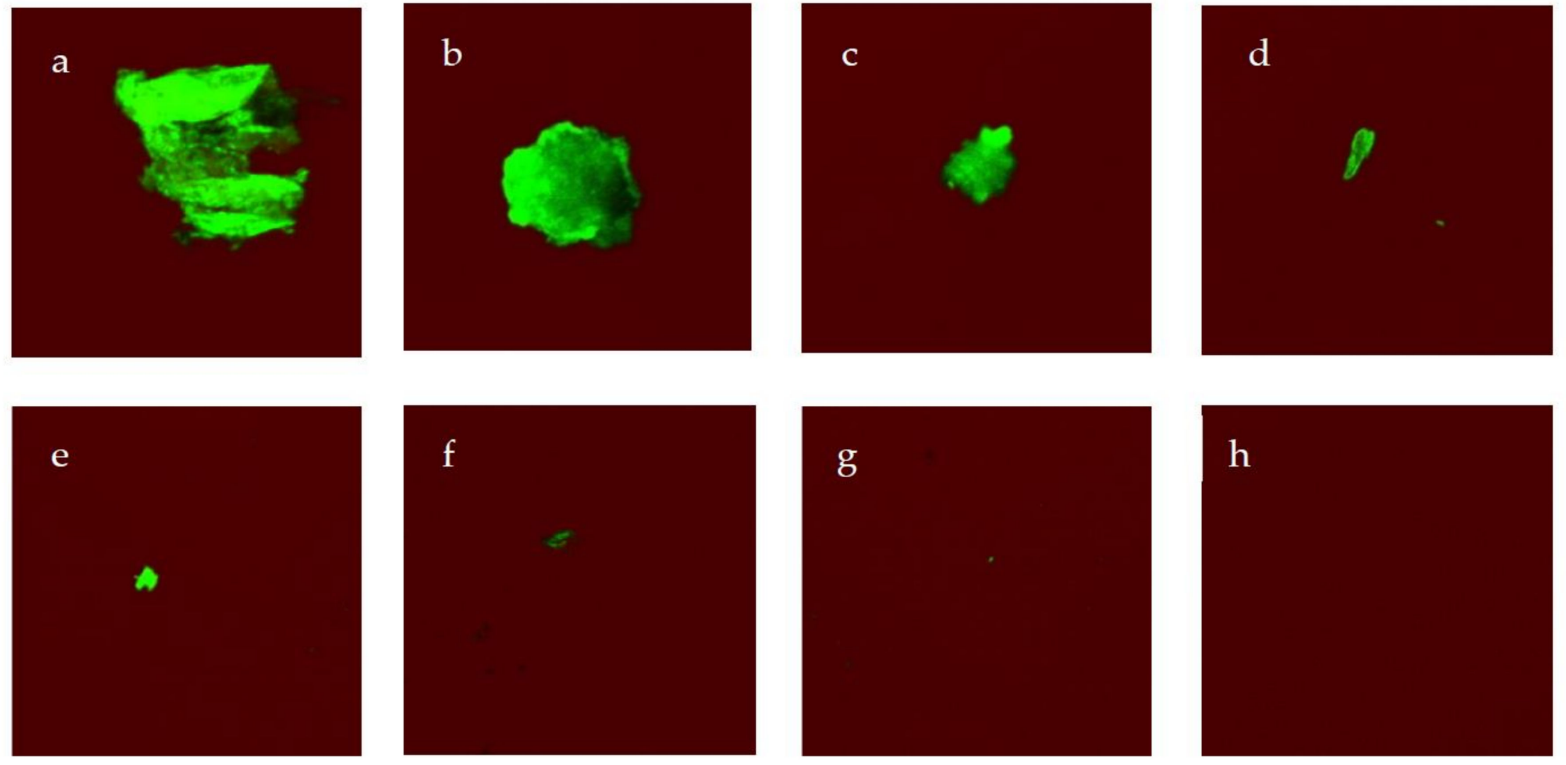
Confocal laser scanning microscopy images of *E. coli* (BL21) cells incubated with the mannose-coated polymer (**a**) 10^9^ CFU mL^−1^; (**b**) 10^8^ CFU mL^−1^; (**c**) 10^7^ CFU mL^−1^; (**d**) 10^6^ CFU mL^−1^; (**e**) 10^5^ CFU mL^−1^; (**f**) 10^4^ CFU mL^−1^; (**g**) 10^3^ CFU mL^−1^; and (**h**) 10^2^ CFU mL^−1^.

To establish the detection limit for this polymer, serial dilutions of the *E. coli* suspension in PBS (10–10^9^ CFU mL^−1^) were prepared and incubated with the polymer for 30 min. After washing out the unbound polymer through thorough washing with PBS solution, the resultant cells were resuspended in PBS and imaged. Microscopic images showed fluorescent clusters of the bacteria can be observed until a concentration of 10^3^ CFU mL^−1^ (Figure 5). Lowering the bacterial concentration below 10^3^ CFU mL^−1^ produced no fluorescent aggregates.

## 3. Materials and Methods

### 3.1. Materials

All chemicals were of analytical grade, sourced from commercial suppliers (Sigma-Aldrich, St. Louis, MO, USA, Fisher Scientific, Hampton, NH, USA) and were used without further purification unless otherwise specified. Phosphate-buffered saline (PBS) at pH 7.4 was prepared by dissolving 1.44 g Na_2_HPO_4_, 0.2 g KCl, 0.24 g KH_2_PO_4_, and 8 g NaCl in purified water (1 L) and autoclaved at 121 °C for 20 min. Absorbance spectra were measured on a Hitachi U-3010 spectrophotometer (Hitachi High-Technologies Corporation, Tokyo, Japan) while fluorescence data were recorded on Hitachi F-2500 fluorescence spectrophotometer. Cell imaging was carried out using a Zeiss LSM 700 confocal microscope (Carl Zeiss, Oberkochen, Germany). *Escherechia coli* (BL21) cells were used throughout this study and were purchased from Transgen Biotech, Beijing, China. Enumeration of bacteria was expressed in CFU mL^−1^.

### 3.2. Polymer Synthesis and Characterization

Synthesis and photophysical measurements of the polymer were carried out and described in our earlier communication [36]. ^1^H-NMR (300 MHz, DMF): δ/ppm 1.23 (s, 18H), 3.5–3.97 (br, 83 H), 4.8 (d, *J* = 1.8 Hz, 0.5 H) 7.6 (s, 4 H). Following characterization, the polymer was stored at 4 °C pending further experimentation with bacterial cells.

### 3.3. Sugar Loading Test

A solution of 60 μg/mL of the mannose-coated polymer in 1 mL DMSO/water mixture (1:1) was mixed with 333 μL of a 5% aqueous solution of phenol to which 1.33 mL of H_2_SO_4_ were added. The reaction mixture was incubated at room temperature for 30 min and its absorbance measured at 490 nm. The increase in absorbance relative to a blank solution containing the same amount of polymer (UV–VIS spectroscopy, Metash, Yuanxi Instrument Ltd., Shanghai, China) was used to quantify the increase in absorbance due to mannose loading. The amount of carbohydrate was determined by comparing with the increase in absorbance from a standard curve generated by testing d-mannose.

### 3.4. Cell Culture and Incubation

BL21 cells were grown in Luria-Bertani (LB) broth medium (medium composition in deionized water (1 L): NaCl (5 g), yeast extract (5 g), and tryptone (10 g)). Incubation of the bacterial cells was carried out overnight at 37 °C with gentle shaking. Bacterial concentration was monitored using an optical density (OD) meter at a wavelength of 600 nm until it reached an OD_600_ of 1. The cells were then centrifuged in tubes at 4000 rpm for 20 min at 4 °C, and washed three times using PBS solution (pH 7.4). An aliquot of the mannose-functionalized polymer (50 µL) was mixed with 5 mL suspension of the cells in PBS, 1 mM CaCl_2_, 1 mM MnCl_2_, 10% glycerol, and incubated at 25 °C with moderate shaking for 40 min.

### 3.5. Optimum Binding Time

BL 21 cells were grown and incubated with the polymer as described in Section 3.4. A portion of the cell-polymer mixture was drawn at 5 min intervals and its fluorescence read using a fluorescence spectrophotometer (FS5 Fluorometer, Edinburg Instruments Ltd., Livingston, UK) until there was no longer any significant change in fluorescence intensity with time.

### 3.6. Sensitivity Test

*E. coli* cells were grown in LB broth medium and incubated overnight at 37 °C with gentle shaking. The cells were then centrifuged at 4000 rpm for 20 min at 4 °C and washed three times using PBS solution (pH 7.4). The cells were then serially diluted (10^9^–10 CFU mL^−1^) using PBS (pH 7.4). To 5 mL of this cell suspension were added 1 mM CaCl_2_, 1 mM MnCl_2_, 10% glycerol, and the polymer. The mixture was incubated at 25 °C with gentle shaking for 30 min. The cells were then centrifuged in tubes at 4000 rpm for 20 min at 4 °C, washed three times using PBS solution (pH 7.4), and re-suspended in PBS.

### 3.7. Cell Imaging

From a portion of the polymer-incubated cells suspended in PBS solution (as prepared in 2.6), 10 µL were drawn, spotted on a glass slide and imaged using a Zeiss LSM 700 confocal laser scanning microscope (Carl Zeiss, Oberkochen, Germany). 

## 4. Conclusions

Specific molecular interaction between mannose and bacteria could allow for mannose-coated polymers to be applied as analytical tools in biosensing. In this study, the utility of the mannose functionalized polymer to detect *E. coli*, expressing functional FimH mannose-specific lectin on their surface was demonstrated. The sugar units displayed on the surface of the polymer retained their functional ability to interact with mannose binding lectin. It was apparent that multivalent interactions are very critical in the detection mechanism. The mannose-coated PPE induced staining and aggregation with *E. coli* with concentrations as low as 10^3^ CFU mL^−1^. Polyvalent interactions between *E. coli* and sugar units on the surface of the polymer were responsible for complex formation. One advantage that the mannose-coated PPE system bears is that it can detect bacteria within a minimum time of 30 min, unlike bacterial conventional detection methods that require selective growth in media, and therefore, take several days to report a result. Coupled with its excellent and intense blue fluorescence as well as high quenching efficiency, this PPE is an attractive sensory material for a number of other analysts.

## Figures and Tables

**Table 1:** Representative photophysical data of polymer P2.

MW/gmol^−1^	Sugar Loading (%)	λ abs/nm	λ em/nm	Stokes Shift (cm^−1^)	ΦF (%)
>14,000	30	393	466.5	73.3	10.8
